# Impact of Legacy Perfluorooctane Sulfonate (PFOS) and Perfluorooctanoate (PFOA) on GABA Receptor-Mediated Currents in Neuron-Like Neuroblastoma Cells: Insights into Neurotoxic Mechanisms and Health Implications

**DOI:** 10.3390/jox14040094

**Published:** 2024-11-12

**Authors:** Laura Lagostena, Davide Rotondo, Davide Gualandris, Antonio Calisi, Candida Lorusso, Valeria Magnelli, Francesco Dondero

**Affiliations:** 1 IBF-CNR, 16149 Genova, Italy; 2 Department of Science and Technological Innovation, Università del Piemonte Orientale, 15121 Alessandria, Italy; davide.rotondo@uniupo.it (D.R.); davide.gualandris@uniupo.it (D.G.); antonio.calisi@uniupo.it (A.C.); candida.lorusso@uniupo.it (C.L.); valeria.magnelli@uniupo.it (V.M.)

**Keywords:** electrophysiology, GABAergic signaling, neurotoxicity, PFAS, persistence, toxicology

## Abstract

Perfluorooctane sulfonate (PFOS) and perfluorooctanoic acid (PFOA) are persistent environmental pollutants, raising concerns due to their widespread presence and disruptive biological effects. These compounds are highly stable, allowing them to bioaccumulate in the environment and living organisms, potentially impacting critical physiological functions such as hormonal balance, immune response, and increasing cancer risk. Despite regulatory restrictions, their pervasive nature necessitates further research into their potential effects on cellular and neuronal function. This study first evaluated the cytotoxic effects of PFOS and PFOA on S1 neuroblastoma cells; a dose-dependent reduction in cell viability was revealed for PFOS, while PFOA exhibited minimal toxicity until millimolar concentrations. We further investigated their potential to modulate GABAergic neurotransmission using patch-clamp electrophysiology. Both PFOS and PFOA caused a significant but reversible reduction in GABA receptor-mediated currents following one-minute pre-treatment. These findings suggest that PFOS and PFOA can interfere with both cellular viability and GABAergic signaling, providing critical insights into their functional impacts and highlighting the need for further investigation into the long-term consequences of PFAS exposure on nervous system health.

## 1. Introduction

Perfluorooctane sulfonate (PFOS) and perfluorooctanoate (PFOA) are notorious environmental contaminants that fall under the extensive category of per- and polyfluoroalkyl substances (PFAS). Renowned for their multiple industrial applications such as aqueous film forming foams (AFFFs), fluoropolymer resins, water- and stain-resistant additives, these chemicals have sparked growing alarm due to their extraordinary persistence in the environment, their ability to bioaccumulate in living organisms, and their potential for long term toxicity including cancer [1]. Of particular concern is the impact of PFOS and PFOA on the nervous system, which is especially susceptible to damage. These compounds can penetrate the blood–brain barrier [2] and accumulate within the brain, in which emerging research indicates they may interfere with normal neuronal function and contribute to neurotoxicity [3]. Although regulatory measures have phased out the production of PFOS and PFOA in many regions, these legacy PFAS compounds remain highly relevant due to their environmental persistence and bioaccumulation potential. While next-generation PFAS compounds are under investigation, these legacy compounds continue to serve as essential models for understanding the persistent impacts of PFAS on human health. Studies across various species, including humans, fish, and polar bears, have documented PFAS accumulation in specific brain regions. Recent findings suggest that PFAS, particularly PFOS, concentrate disproportionately in protein-rich regions of the brain, such as the hypothalamus [4], which is critical for neuroendocrine regulation. This selective accumulation implies that even low environmental exposures may lead to significant buildup in key neuroendocrine centers, potentially disrupting GABAergic signaling and other essential physiological processes.

Furthermore, recent studies have detected relatively high concentrations of PFOA and total PFAS in the brains of potentially exposed individuals [5], indicating that chronic, low-dose environmental exposures can result in meaningful accumulation over time. Given this context, our study used controlled, elevated doses of PFAS to elucidate potential acute effects on GABAergic signaling, providing a mechanistic foundation that could relate to cumulative impacts in chronic exposure scenarios.

One crucial aspect of neuronal function involves gamma-aminobutyric acid (GABA) receptors, which play a pivotal role in maintaining the excitatory–inhibitory balance in the brain. GABA receptors, particularly the GABA_A_ subtype, mediate inhibitory synaptic transmission and are essential for various neurological processes, including anxiety modulation, muscle relaxation, and seizure prevention. Disruption of GABA_A_ receptor-mediated currents can, therefore, have profound implications for brain function and overall health. GABA_A_ receptors are crucial components of the nervous system, functioning as ligand-gated chloride channels that play a key role in inhibitory neurotransmission. These receptors are composed of five subunits arranged in a pentameric structure, with each subunit drawn from a diverse set of possibilities. In humans, researchers have identified 19 distinct genes that encode the subunits of GABA_A_ receptors. These include six α (1–6), three β (1–3), three γ (1–3), three ρ (1–3), and one each for δ, ε, π, and θ subunits [6,7]. The vast diversity observed in GABA_A_ receptors stems from the multiple ways these subunits can combine, which is further enhanced by the alternative splicing of several genes. This alternative splicing process allows a single gene to produce multiple different subunit variants, thereby significantly expanding the potential combinations and functional diversity of the receptors. This diversity is critical, as it enables GABA_A_ receptors to fulfill a wide range of functions in various regions of the brain and to modulate neuronal activity in response to different physiological, and pharmacological stimuli [8,9,10]. The α1β2γ2 subtype is the predominant form in the mammalian nervous system, representing a significant majority. It is estimated that this specific configuration may constitute as much as 60% of all GABA_A_ receptors [9], highlighting its crucial role in functioning and regulation of neural activity [11]. GABA_A_ receptors are ligand-gated chloride channels, made by pentameric combination of different subunits. GABA_A_ receptor subunits share a common structure. Each mature subunit consists of approximately 450 amino acid residues and includes several key regions: an N-terminal region, a large hydrophilic extracellular domain (ECD), and four hydrophobic transmembrane domains (TMD: TM1–TM4). The TM2 domain is thought to form the pore of the chloride channel, while an intracellular domain (ICD) located between TM3 and TM4 serves as a critical site for protein interactions and post-translational modifications that regulate receptor activity [12,13]. The neurotransmitter GABA, along with psychotropic drugs like benzodiazepines (BZDs), binds to the N-terminal at the α–β and α–γ interfaces, respectively. Additionally, neurosteroids and anesthetics such as barbiturates interact within the TMD of the α and β subunits [14,15]. Several GABAergic insecticides, despite their diverse chemical structures, act as noncompetitive antagonists by binding to the same GABA receptor site located in the TM2 domain. Compounds such as β-endosulfan, lindane, and fipronil, along with botanical agents like picrotoxinin, target the chloride channel pore of the GABA receptor, blocking ion flow and leading to neural hyperexcitation, which is critical for insecticidal action and human neurotoxicity [16].

This study aimed to investigate the impact of PFOS and PFOA on GABA receptor-mediated currents in neuron-like neuroblastoma cells, providing insights into the neurotoxic mechanisms of these pollutants. For this purpose, we utilized the S1 neuroblastoma cell line, a specialized derivative of the SK-N-BE(2) cell line, specifically developed to express functional GABA_A_ receptors. Unlike standard SH-SY5Y cells, which do not exhibit GABA-evoked responses [17], the S1 line is especially suited for examining PFAS effects on GABAergic signaling, offering a controlled study model for preliminary toxicology assessment [18]. This research directly reveals how legacy PFAS affect neuron-like cells, representing a major advancement in our understanding. By examining the interactions between PFOS, PFOA, and GABA_A_ receptors, we seek to uncover how these interactions may disrupt neuronal activity. This knowledge will help elucidate the broader health implications of exposure to these widespread environmental contaminants. The study not only deepens our understanding of PFAS-induced neurotoxicity but also highlights the urgent need for stringent regulatory measures to limit exposure and protect public health. The findings are expected to prompt policy changes and interventions aimed at reducing the prevalence of these harmful substances, thereby safeguarding both human health and environmental integrity.

## 2. Materials and Methods

### 2.1. Cell Culture

S1 neuroblastoma cells were maintained as reported in [18]. Briefly, cells were grown in RPMI 1640 medium supplied with 10% FBS, 2 mM L-Glu, 100 μg/mL penicillin–streptomycin, 200 μg/mL geneticin sulfate (G418), and kept at 37 °C in a 5% CO_2_ incubator. All chemicals were purchased from Merck (Sigma-Aldrich, Milan, Italy).

### 2.2. Cytotoxicity Assay

Cells were seeded in 96 multiwell plates (Greiner) at a density of 5 × 10^3^ cells per well in complete medium as described in Section 2.1. Once settled, the medium was removed and replaced with fresh medium containing varying concentrations of PFOA and PFOS to assess their impact on cell viability. The plates were incubated for 72 h at 37 °C in a 5% CO_2_ atmosphere to allow for cell adhesion, proliferation, and PFAS exposure. Following this incubation, resazurin was added to each well at a concentration of 0.1 mg/mL. The plates were further incubated for 2 h under the same conditions, after which fluorescence was measured using a Tecan Infinite 200 Pro fluorimeter (Tecan Group Ltd., Männedorf, Switzerland) (excitation 535 nm, emission 590 nm) to quantify the reduction of resazurin to resorufin, indicating cell viability [19] For statistics, data were evaluated for normality and homoscedasticity and analyzed using one-way analysis of variance (ANOVA), followed by Holm–Sidak’s multiple comparisons test using GraphPad Prism^TM^ 9 (GraphPad Software, San Diego, CA, USA).

### 2.3. Electrophysiological Recordings

S1 neuroblastoma cells were plated in 3.5 cm Petri dishes and submerged in a bath of extracellular solution containing the following (in mM): 140 NaCl, 5.4 KCl, 2 CaCl_2_, 1 MgCl_2_, 10 HEPES, and 10 glucose (pH adjusted to 7.4 with NaOH). Observations were performed under an inverted microscope (IMT-2, Olympus Life Science, Milan, Italy) using a 40× objective. Whole-cell patch-clamp recordings were conducted at room temperature (22–25 °C) using an Axopatch 200A amplifier (Molecular Devices, San Jose, CA, USA). Patch pipettes were fabricated from borosilicate glass capillaries (Hilgenberg, Malsfeld, Germany), pulled using a vertical pipette puller (PIP6, HEKA, Lambrecht, Germany), and filled with internal solution containing the following (in mM): 142 KCl, 2 MgCl_2_, 2 EGTA, and 10 HEPES (pH adjusted to 7.3 with KOH). Final pipette resistance ranged from 3–5 MΩ.

After establishing a stable whole-cell configuration, cells were clamped at a holding potential (Vh) of −80 mV. Voltage-gated currents were elicited by applying voltage pulses ranging from −100 mV to +100 mV in 20 mV increments, each lasting for 20 s. GABA-evoked currents were activated by brief agonist application via a gravity-driven perfusion system localized near the patched cell. Recordings were acquired using a custom program, GePulse (http://users.ge.ibf.cnr.it/pusch/programs-mik.htm) (accessed on 10 September 2024) on, and whole-cell currents were low-pass filtered at 2 kHz and digitized at 20 kHz.

GABA stock solution (in distilled water), picrotoxin (PTX, in DMSO), and PFOS or PFOA (in DMSO or isopropanol, respectively) were freshly prepared and added to the saline solution, ensuring the final concentration of DMSO or isopropanol remained at 0.02% (*v*/*v*) prior to each experiment. This careful preparation maintained the integrity and consistency of the experimental conditions. GABA, PTX, and PFAS were rapidly applied to patched cells using the gravity-driven perfusion system, followed by a 1 min washout period between applications to allow for receptor recovery and minimize desensitization effects. To prevent cross-contamination, PFOS and PFOA were tested on different S1 cells from separate Petri dishes.

Acquired data were analyzed offline using Clampfit 10.7 (Molecular Devices, USA) and SigmaPlot (Systat Software Inc., San Jose, CA, USA). Data are expressed as mean ± standard error, and statistical significance was determined using a paired *t*-test.

## 3. Results

### 3.1. Acute Toxicity of PFAS in S1 Cells

**Figure 1:**
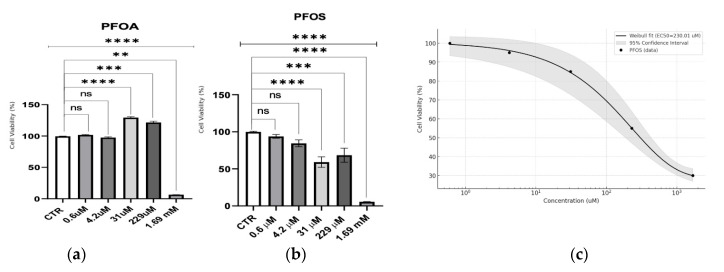
Cytotoxicity assessment using the Alamar Blue test on S1 neuroblastoma cells. (**a**) Dose-dependent effects of PFOA on cell viability, expressed as a percentage relative to the control (CTR). (**b**) Dose-dependent effects of PFOS on cell viability, expressed as a percentage relative to the control (CTR). The upper line represents the outcome of the statistical test, where the control group (CTR) was compared to all treated samples, regardless of concentration level. Statistical significance is indicated as follows: ns, not significant (*p* > 0.05), ** *p* < 0.01, *** *p* < 0.001, **** *p* < 0.0001. (**c**) Weibull dose-response curve for PFOS, showing the fit (solid black line) with an estimated EC50 of 230.01 µM. The 95% confidence intervals (shaded gray area) are shown, along with the averaged data points (black circles).

This study assessed the cytotoxic effects of PFOA and PFOS across a range of PFAS concentrations (Figure 1) in neuron-like S1 neuroblastoma cells. PFOA displayed a threshold response, with minimal toxicity observed until millimolar concentrations. A stimulatory effect was noted at 229 µM, followed by a sharp drop in viability only at 1.69 mM, suggesting that acute toxicity for PFOA is virtually absent at lower doses. In contrast, PFOS exhibited a dose-dependent cytotoxic response. A Weibull regression analysis for PFOS indicated a significant reduction in cell viability across a broader concentration range, with an estimated EC50 of 230.01 ± 26.31 µM, highlighting its higher acute toxicity compared to PFOA.

### 3.2. Electrophysiological Characterization of S1 Neuron-Like Neuroblastoma Cells

After verifying the effect of PFAS on the survival of neuron-like cells, we investigated whether these substances could exert an effect on synaptic transmission on GABA receptors.

**Figure 2:**
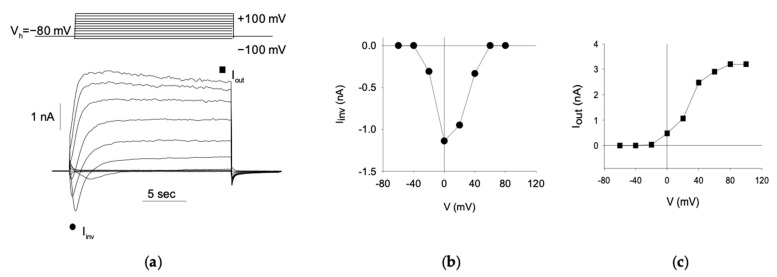
Voltage-activated currents in differentiated S1 neuroblastoma cells. (**a**) Representative current responses (**bottom**) evoked by the voltage protocol (illustrated in the top panel) applied to a patched S1 cell. (**b**) current–voltage (I–V) relationship of the inward currents (value taken at the peak value, ●) recorded from the cell shown in A. (**c**) I–V relationship of the outward currents (at the steady-state level, ■) for the same cell.

First, to confirm the neuron-like differentiation of S1 neuroblastoma cells, we investigated the presence of voltage-dependent currents. Voltage steps of 20 mV were applied every 20 s, ranging from −100 mV to +100 mV, starting from a holding potential (Vh) of −80 mV (Figure 2a, top). This protocol revealed both inward and outward currents (Figure 2a, bottom) in all patched cells (330/330, 100%). The inward currents are indicative of Na^+^-channel activity, demonstrating a typical voltage-dependent activation and inactivation pattern (Figure 2b), while the outward currents suggest K^+^-channel activity, characterized by a voltage-dependent activation that is sustained over the voltage steps (Figure 2c).

Since an exhaustive characterization of voltage-gated currents in S1 cells has been provided by Gavazzo et al. [18], further investigation was not pursued. These findings confirm that S1 neuroblastoma cells have differentiated into an excitatory neuron-like phenotype.

**Figure 3:**
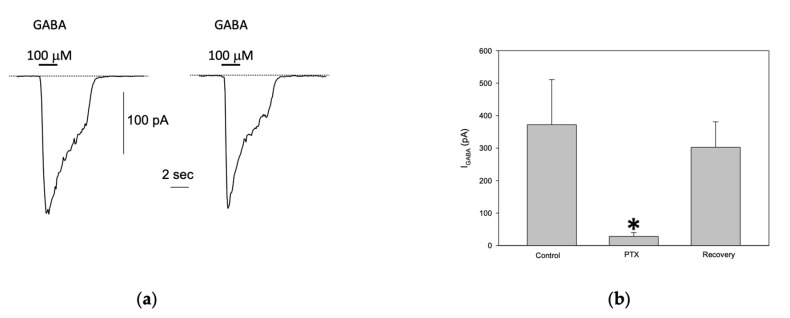
GABA_A_ activated currents in differentiated S1 neuroblastoma cells. (**a**) Two successive applications of 100 μM GABA, administered 1 min apart to a patched S1 cell, elicited stable and repetitive inward current responses. (**b**) Bath application of PTX significantly and reversibly reduced the GABA-evoked currents (* *p* < 0.05).

To assess the presence of functional GABAergic receptors in S1 cells, rapid application of 100 μM GABA to patched cells, held at −80 mV, elicited an inward current carried by Cl^−^ ions following the electrochemical gradient. The mean current amplitude was 249 ± 29.5 pA (ranging from 23 to 1314 pA), which aligns well with previous findings [18]. However, only 27% (89/330) of the cells responded to GABA, suggesting a significant reduction in the proportion of S1 cells expressing functional GABA_A_ receptors. Despite this, in cells that did respond, the GABA-evoked current was reproducible and stable across multiple stimulations (Figure 3a). This indicates the reliability of GABA-induced responses in cells expressing functional GABA_A_ receptors. Additionally, picrotoxin (PTX, 100 μM), a known GABA_A_ receptor antagonist, significantly and reversibly inhibited the GABA-evoked currents (control, 372 ± 139 pA; PTX, 28.17 ± 12.1 pA; recovery, 302.5 ± 78.36, *n* = 6, Figure 3b), confirming that the observed inward currents were indeed mediated by GABA_A_ receptors, as previously reported [18].

### 3.3. Effect of PFOS and PFOA on GABA-Evoked Currents in Neuron-Like S1 Neuroblastoma Cells

To determine whether the two PFAS compounds influence baseline current in neuron-like cells, a localized application of sublethal PFOS or PFOA concentrations—10 μM—was administered to patched S1 cells. The results showed that neither compound induced any detectable currents, nor were there any observable effects on the baseline current (data not shown), consistent with findings reported by Tukker et al. [20]. This lack of response strongly suggests that PFOS and PFOA, when applied alone at this concentration, do not have an immediate or direct impact on the baseline electrical activity of these cells.

**Figure 4:**
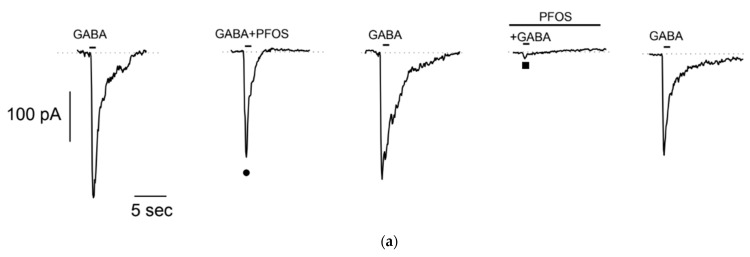

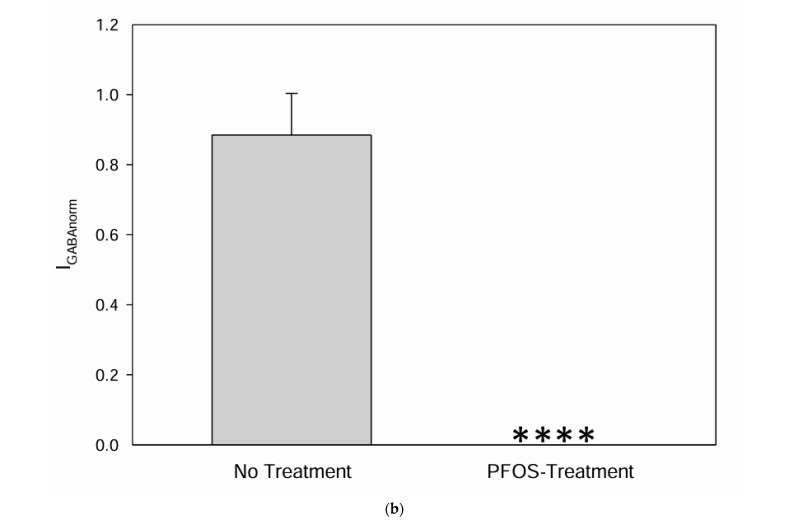
Effect of PFOS on GABA-activated currents in differentiated S1 neuroblastoma cells. (**a**) Representative effect of PFOS-modulation on GABA-current with (■) or without (●) PFOS-pretreatment from the same patched cell. Reversible almost complete current reduction was observed when S1 cells were previously exposed to PFOS. (**b**) Effect of PFOS on normalized GABA-amplitude (mean ± SE, *n* = 5) showing significant inhibition (**** *p* < 0.0001) when cells were pre-treated with PFOS.

In order to investigate if PFOS is able to modulate GABA-evoked currents, we co-applied GABA with PFOS at 10 μM, a concentration previously shown to be effective in injected oocytes [20]. In GABA-responding S1 cell, brief co-application of PFOS showed no significant effect on GABA-evoked current (Figure 4a •). However, when S1 cell was pretreated with PFOS for 1 min, current amplitude was almost completely suppressed (Figure 4a ■). After a 1 min washout, the current recovered, indicating that PFOS block on GABA-dependent current is reversible. Similar results were observed in *n* = 5 cells. To reduce data variability due to scattering of GABA-evoked current amplitude, responses in the presence of PFOS were normalized to responses recorded without it (no treatment: 0.88 ± 0.12; pre-treatment: 6.8 × 10^−5^ ± 5.12 × 10^−5^, see plot on Figure 4b).

**Figure 5:**
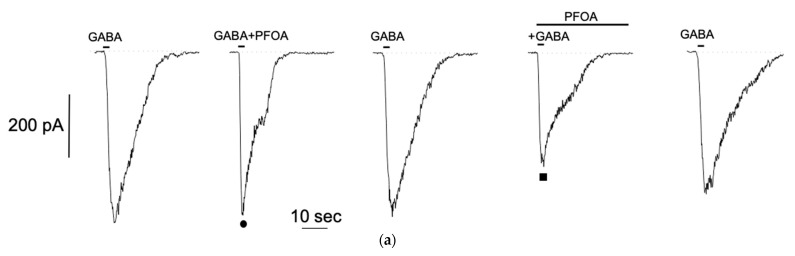

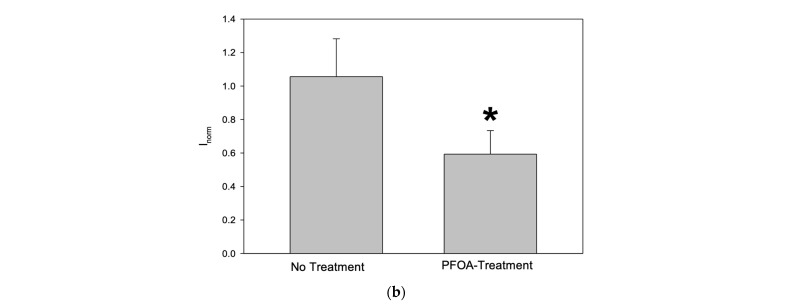
Effect of PFOA on GABA-activated currents in differentiated S1 neuroblastoma cells. (**a**) Representative effect of PFOA-modulation on GABA-current with (■) or without (●) PFOA-pretreatment from the same patched cell. Reversible current reduction was observed when S1 cells were previously exposed to PFOA. (**b**) Effect of PFOA on normalized GABA-amplitude (mean ± SE) showing significant inhibition (* *p* < 0.05) when cells were pre-treated.

The same experimental protocol was applied to GABA-responsive cells to investigate the effects of PFOA. Like PFOS, PFOA reduced the GABA-evoked current amplitude only when S1 cells were pretreated (Figure 5a). Although the block of GABA-dependent current was less pronounced (no treatment normalized current: 1.05 ± 0.23, *n* = 5; pre-treatment: 0.6 ± 0.14, *n* = 6, Figure 5b), it was still significant, and reversible as seen for the PFOS.

## 4. Discussion

The results of this study provide compelling evidence that both PFOS and PFOA significantly alter GABAA receptor-mediated currents in neuron-like neuroblastoma S1 cells, revealing important insights into the neurotoxic mechanisms of these persistent pollutants. The choice to utilize the S1 neuroblastoma cell line for this study is grounded in the principles of non-animal testing models, which align with the 3Rs framework (Replacement, Reduction, and Refinement) aimed at minimizing reliance on animal experimentation. Neuroblastoma cells provide a relevant platform for studying the neurotoxic effects of PFAS compounds, as they retain key neuronal characteristics and exhibit similar physiological responses to excitatory and inhibitory signaling. Moreover, traditional methods relying on animal testing are being replaced by 3R principle-based alternatives, emphasizing in vitro test methods that consider broader chemical–biological interactions. Recent advances, as discussed by Giusy del Giudice et al. [21], highlight the importance of leveraging multiple informational levels to strengthen the understanding of biological responses, ultimately enhancing the predictive capacity of in vitro methods. By utilizing the S1 neuroblastoma cell line, we can effectively explore the mechanisms of PFAS-induced neurotoxicity while adhering to ethical considerations and improving the reliability of our findings.

The reduction in GABA-evoked currents following pre-treatment with PFOS and PFOA highlights a potentially critical pathway by which these compounds exert their neurotoxic effects, specifically through the modulation of inhibitory neurotransmission.

While the 10 μM concentration used in this study may appear elevated compared to typical environmental exposures, data from both community and occupational studies strongly support its relevance. Evidence suggests that PFAS accumulation in the brain could approach levels observed in our findings, especially under chronic environmental conditions. Recent analyses have reported average PFOA concentrations close to 160 ng/g in the hypothalamus of deceased individuals from areas near contamination sources, with variability indicating that brain levels may reach two to three times higher in certain individuals [5].

Remarkably, a nearly 1:1 brain-to-blood concentration ratio observed in some cases suggests a direct proportionality in PFAS retention between serum and brain tissue [4,5]. In communities near contamination sites, mean serum PFOA levels of approximately 1 µM (423 ng/mL) have been reported among residents, with even higher levels observed in children aged 2–5 approaching 1 ppm as maximum value [22]. These findings underscore that even non-occupationally exposed individuals in highly contaminated areas can experience elevated serum PFOA concentrations. In occupational settings, exposure levels are even significantly higher. Median serum PFOA levels of up to 2.88 ppm have been documented among workers in specific roles within fluoropolymer manufacturing plants, with maximum values reaching 59.4 ppm (148.5 µM) [23]. Additionally, historical data indicate that serum PFOA levels could reach up to 92 ppm (230 µM) in highly exposed industrial workers, reflecting cumulative exposure over extended periods due to direct handling of PFAS-containing materials [24].

These data illustrate a continuum of exposure, from elevated community levels to extreme concentrations in occupational settings, supporting the relevance of our chosen 10 µM concentration. By using this level in vitro, we simulate acute effects that provide insights into potential neurotoxic mechanisms of PFOA and PFOS. This concentration models effects likely present in occupationally exposed individuals and provides a mechanistic basis for understanding cumulative impacts from long-term, low-dose exposure in the general population. Thus, our experimental approach serves as a valuable model for investigating PFAS-induced neurotoxicity in both environmental and occupational contexts.

First, it must be pointed out that, due to the GABA amplitude scattering and a limited number of GABA-expressing cells, we used GABA at a very high concentration (100 µM) to increase the probability of finding responsive cells. We are aware that this concentration is saturating, that some other effects of PFAS could be masked, and that it is necessary to conduct experiments with lower doses in a more suitable cellular model to better understand PFAS effects. Nevertheless, our results are in good agreement with Tukker’s data [20], showing a PFOS/PFOA-dependent reduction of current in GABA-expressing oocytes. Moreover, they claim that both PFAS are non-competitive GABA antagonists. Similarly, our findings indicate that PFOS and PFOA do not directly induce currents or alter baseline electrical activity in the S1 neuroblastoma cells when applied alone. This suggests that their neurotoxic effects may not stem from direct interaction with the GABA_A_ receptor in the absence of the neurotransmitter, supporting the hypothesis of an indirect effect on the receptor. However, the significant reduction in GABA-evoked currents following pre-exposure to these PFAS compounds suggests that PFOS and PFOA may interfere with the receptor’s ability to respond to GABA, potentially by altering receptor conformation, hindering receptor function, or affecting the receptor’s interaction with modulatory binding sites known to be present in the GABAA receptors. There is compelling evidence suggesting that the neurotoxic effects of PFAS may result from their accumulation in cell membranes [25], as these compounds tend to integrate into cell membranes and other lipid bilayers due to their lipophilic nature [3,26,27,28]. Moreover, PFAS can alter plasma membrane potential and intracellular pH level [29]. However, the effects observed in our study are unlikely to be exclusively attributed to these mechanisms. We observed no significant impact on baseline current, and the relative brief 1 min exposure followed by rapid washout makes membrane accumulation an improbable cause for the observed outcomes. The effect on membrane potentials seems unlikely, as adding just 10 micromolar of negative charge on the extracellular side would not be sufficient to cause a complete block or even a 60% reduction of GABA-induced current. Furthermore, if the effect were purely charge-dependent, both PFOS and PFOA would produce identical outcomes due to their shared negative charge. Additionally, there was no observed impact on the baseline current. However, the potential impact of PFAS on cell membranes cannot be definitively excluded. While our findings suggest that membrane accumulation may not be the primary mechanism, especially given the brief exposure and rapid washout, it remains a possibility that warrants further investigation

In our neuron-like S1 cells, both PFAS effects were reversible within seconds upon washout. However, in GABA-expressing oocytes, PFOS-induced inhibition was poorly reversible, suggesting a stronger and more persistent interaction with the receptor in this model [20]. This discrepancy could be ascribed to differences in biological systems, as oocytes express recombinant GABA_A_ receptors with controlled subunit composition, which may lack the accessory proteins or native conditions present in neuronal cells [20]. Additionally, the subunit composition of GABA_A_ receptors can vary significantly between models, potentially affecting PFAS binding affinity and reversibility. Nonetheless, the reversibility of PFAS-dependent inhibition suggests that the interaction between PFOS or PFOA and GABA_A_ receptors is not permanently damaging but rather a temporary interference, particularly when PFAS exposure is brief and localized. This reversible nature also raises concerns about the potential for chronic exposure to cause cumulative neurotoxic effects, where repeated or prolonged exposure might lead to lasting disruptions in inhibitory signaling.

GABA_A_ receptors play a crucial role in maintaining the excitatory–inhibitory balance in the central nervous system. Disruptions to this balance, such as those caused by PFOS and PFOA, can have far-reaching consequences, potentially leading to increased neuronal excitability, impaired neural circuits, and the onset of neurological disorders. The fact that PFOS and PFOA can suppress GABA-evoked currents suggests that their presence could contribute to altered neural excitability and function, particularly under conditions of prolonged exposure where recovery between exposures may be incomplete. These findings are particularly concerning given the widespread and persistent nature of PFOS and PFOA in the environment and their ability to bioaccumulate in human tissues, including the brain.

Evidence from animal models further underscores the relevance of our in vitro exposure levels. For example, PFOS has been shown to accumulate to concentrations as high as 17 µM in the hippocampus of mice following sub-chronic exposure [30]. Such accumulation in the hippocampus, a brain region crucial for memory and learning, suggests that our chosen in vitro concentration mirrors real-world levels that PFOS can achieve in specific neural structures. This accumulation aligns with our findings on GABAergic suppression, indicating that PFOS can disrupt normal neurotransmission at concentrations observable in vivo. Additionally, research in hippocampal neurons [31] demonstrates that PFOS can enhance excitatory signaling by increasing the frequency and amplitude of excitatory miniature postsynaptic currents and amplifying field excitatory postsynaptic potentials. The combination of increased excitatory drive and suppressed inhibitory control suggests a potential shift toward hyperexcitability in hippocampal circuits, a change that could impair synaptic plasticity mechanisms essential for cognitive function, such as long-term potentiation and long-term depression. This pro-excitatory state may lead to excitotoxicity under prolonged exposure, aligning with epidemiological evidence linking PFAS exposure to cognitive deficits, developmental delays, and other neurological conditions [3,32,33]. Collectively, these findings support the neurotoxic potential of PFOS and reinforce the need for examining its effects on GABAergic and excitatory systems in the context of environmental exposure.

The study underscores the broader health implications of exposure to PFOS and PFOA, particularly regarding their potential to disrupt critical neurobiological processes. The observed effects on GABA_A_ receptor-mediated currents provide a mechanistic link between PFAS exposure and neurotoxic outcomes, supporting the hypothesis that these compounds could contribute to the development of neurological disorders.

This research highlights the need for further studies to explore the long-term effects of chronic PFAS exposure on GABAergic neurotransmission and overall brain health. Moreover, the findings call attention to the urgent need for stringent regulatory measures to limit PFAS exposure. The potential for these compounds to interfere with key neurotransmitter systems suggests that current exposure levels, even if deemed safe by existing standards, could pose significant risks to public health, particularly for vulnerable populations such as children and individuals with pre-existing neurological conditions.

## 5. Perspectives

Looking forward, it is essential to deepen our understanding of the mechanisms underlying PFAS-induced inhibition of GABA_A_ receptor function. Future studies should aim to identify the specific sites of interaction between PFOS/PFOA and the GABA_A_ receptor. Conducting molecular docking studies could provide valuable insights into how these compounds interact at the molecular level with the receptor’s binding sites or transmembrane regions. Such computational approaches can predict potential binding sites and modes, guiding experimental efforts to confirm these interactions.

Additionally, exploring the effects of a broader range of PFAS compounds on GABA_A_ receptor function is crucial. Given the structural diversity within the PFAS family, including congeners and classes that are not yet restricted or banned, testing other PFAS could reveal whether the inhibitory effects observed with PFOS and PFOA are common to other related compounds or are specific legacy ones. This would enhance our understanding of the structure–activity relationships governing PFAS interactions with GABA_A_ receptors and identify potential risks associated with other PFAS substances that are currently in use.

From a public health perspective, our findings highlight the importance of revisiting safety standards and regulatory policies concerning PFAS exposure. Considering their persistent nature and bioaccumulative properties, even low-level exposure could have significant health implications over time. Collaborative efforts between scientists, policymakers, and industry stakeholders are necessary to develop strategies for reducing PFAS emissions, remediating contaminated environments, and limiting human exposure.

Finally, investigating potential therapeutic interventions to mitigate PFAS-induced neurotoxicity is an important area for future research. Identifying compounds or treatments that can counteract the inhibitory effects of PFAS on GABA_A_ receptors may offer protective strategies for individuals at risk of exposure.

## 6. Conclusions

This study demonstrates that PFOS and PFOA significantly inhibit GABA_A_ receptor-mediated currents in neuron-like neuroblastoma S1 cells. These findings suggest that PFAS compounds can disrupt inhibitory neurotransmission, potentially shifting the excitatory–inhibitory balance in the brain and increasing the risk of neurological disorders associated with excitotoxicity and cognitive deficits. The relevance of our findings is further underscored by reports of legacy PFAS accumulation in brain regions like the hippocampus at levels comparable to those used in our experiments. Given the widespread presence of these bioaccumulative compounds in the environment, there is an urgent need to reassess safety standards and minimize human exposure to PFAS to protect neurological health.

## Data Availability

The original contributions presented in the study are included in the article, further inquiries can be directed to the corresponding authors.

## References

[1] Zahm S., Bonde J.P., Chiu W.A., Hoppin J., Kanno J., Abdallah M., Blystone C.R., Calkins M.M., Dong G.H., Dorman D.C. (2024). Carcinogenicity of perfluorooctanoic acid and perfluorooctanesulfonic acid. Lancet Oncol..

[2] Yu Y., Wang C., Zhang X., Zhu J., Wang L., Ji M., Zhang Z., Ji X.M., Wang S.L. (2020). Perfluorooctane sulfonate disrupts the blood brain barrier through the crosstalk between endothelial cells and astrocytes in mice. Environ. Pollut..

[3] Brown-Leung J.M., Cannon J.R. (2022). Neurotransmission Targets of Per- and Polyfluoroalkyl Substance Neurotoxicity: Mechanisms and Potential Implications for Adverse Neurological Outcomes. Chem. Res. Toxicol..

[4] Starnes H.M., Rock K.D., Jackson T.W., Belcher S.M. (2022). A critical review and meta-analysis of impacts of per-and polyfluorinated substances on the brain and behavior. Front. Toxicol..

[5] Di Nisio A., De Toni L., Sabovic I., Guidolin D., Dall’Acqua S., Sfriso M.M., Rocca M.S., De Filippis V., Foresta C., Garolla A. (2021). Impairment of human dopaminergic neurons at different developmental stages by perfluoro-octanoic acid (PFOA) and differential human brain areas accumulation of perfluoroalkyl chemicals. Environ. Res..

[6] Sieghart W., Fuchs K., Tretter V., Ebert V., Jechlinger M., Hoger H., Adamiker D. (1999). Structure and subunit composition of GABA(A) receptors. Neurochem. Int..

[7] Steiger J.L., Russek S.J. (2004). GABAA receptors: Building the bridge between subunit mRNAs, their promoters, and cognate transcription factors. Pharmacol. Ther..

[8] Ghit A., Assal D., Al-Shami A.S., Hussein D.E.E. (2021). GABA(A) receptors: Structure, function, pharmacology, and related disorders. J. Genet. Eng. Biotechnol..

[9] Rudolph U., Knoflach F. (2011). Beyond classical benzodiazepines: Novel therapeutic potential of GABAA receptor subtypes. Nat. Rev. Drug Discov..

[10] Sigel E., Baur R., Trube G., Mohler H., Malherbe P. (1990). The effect of subunit composition of rat brain GABAA receptors on channel function. Neuron.

[11] Sallard E., Letourneur D., Legendre P. (2021). Electrophysiology of ionotropic GABA receptors. Cell Mol. Life Sci..

[12] Chen Z.W., Olsen R.W. (2007). GABAA receptor associated proteins: A key factor regulating GABAA receptor function. J. Neurochem..

[13] Jacob T.C., Moss S.J., Jurd R. (2008). GABA(A) receptor trafficking and its role in the dynamic modulation of neuronal inhibition. Nat. Rev. Neurosci..

[14] Sigel E., Luscher B.P. (2011). A closer look at the high affinity benzodiazepine binding site on GABAA receptors. Curr. Top Med. Chem..

[15] Wang M. (2011). Neurosteroids and GABA-A Receptor Function. Front. Endocrinol..

[16] Chen L., Durkin K.A., Casida J.E. (2006). Structural model for γ-aminobutyric acid receptor noncompetitive antagonist binding: Widely diverse structures fit the same site. Proc. Natl. Acad. Sci. USA.

[17] Nikonorov I.M., Blanck T.J., Recio-Pinto E. (2003). G-protein Activation Decreases Isoflurane Inhibition of N-type Ba^2+^ Currents. Anesthesiology.

[18] Gavazzo P., Vella S., Marchetti C., Nizzari M., Cancedda R., Pagano A. (2011). Acquisition of neuron-like electrophysiological properties in neuroblastoma cells by controlled expression of NDM29 ncRNA. J. Neurochem..

[19] Rampersad S.N. (2012). Multiple applications of Alamar Blue as an indicator of metabolic function and cellular health in cell viability bioassays. Sensors.

[20] Tukker A.M., Bouwman LM S., van Kleef R., Hendriks H.S., Legler J., Westerink R.H.S. (2020). Perfluorooctane sulfonate (PFOS) and perfluorooctanoate (PFOA) acutely affect human alpha(1)beta(2)gamma(2L) GABA(A) receptor and spontaneous neuronal network function in vitro. Sci. Rep..

[21] Del Giudice G., Migliaccio G., D’Alessandro N., Saarimäki L.A., Torres Maia M., Annala M.E., Leppänen J., Möbus L., Pavel A., Vaani M. (2023). Advancing chemical safety assessment through an omics-based characterization of the test system-chemical interaction. Front. Toxicol..

[22] Emmett E.A., Shofer F.S., Zhang H., Freeman D., Desai C., Shaw L.M. (2006). Community exposure to perfluorooctanoate: Relationships between serum concentrations and exposure sources. J. Occup. Environ. Med..

[23] Woskie S.R., Gore R., Steenland K. (2012). Retrospective exposure assessment of perfluorooctanoic acid serum concentrations at a fluoropolymer manufacturing plant. Ann. Occup. Hyg..

[24] Olsen G.W., Burris J.M., Ehresman D.J., Froehlich J.W., Seacat A.M., Butenhoff J.L., Zobel L.R. (2007). Half-life of serum elimination of perfluorooctanesulfonate, perfluorohexanesulfonate, and perfluorooctanoate in retired fluorochemical production workers. Environ. Health Perspect..

[25] Zhao L., Teng M., Zhao X., Li Y., Sun J., Zhao W., Ruan Y., Leung KM Y., Wu F. (2023). Insight into the binding model of per- and polyfluoroalkyl substances to proteins and membranes. Environ. Int..

[26] Cao Y., Ng C. (2021). Absorption, distribution, and toxicity of per- and polyfluoroalkyl substances (PFAS) in the brain: A review. Environ. Sci. Process. Impacts.

[27] Harada K.H., Ishii T.M., Takatsuka K., Koizumi A., Ohmori H. (2006). Effects of perfluorooctane sulfonate on action potentials and currents in cultured rat cerebellar Purkinje cells. Biochem. Biophys. Res. Commun..

[28] Naumann A., Alesio J., Poonia M., Bothun G.D. (2022). PFAS fluidize synthetic and bacterial lipid monolayers based on hydrophobicity and lipid charge. J. Environ. Chem. Eng..

[29] Kleszczynski K., Skladanowski A.C. (2009). Mechanism of cytotoxic action of perfluorinated acids. I. alteration in plasma membrane potential and intracellular pH level. Toxicol. Appl. Pharmacol..

[30] Austin M.E., Kasturi B.S., Barber M., Kannan K., MohanKumar P.S., MohanKumar S.M. (2003). Neuroendocrine effects of perfluorooctane sulfonate in rats. Environ. Health Perspect..

[31] Liao C.Y., Li X.Y., Wu B., Duan S.M., Jiang G.B. (2008). Acute enhancement of synaptic transmission and chronic inhibition of synaptogenesis induced by perfluorooctane sulfonate through mediation of voltage-dependent calcium channel. Environ. Sci. Technol..

[32] Panieri E., Baralic K., Djukic-Cosic D., Buha Djordjevic A., Saso L. (2022). PFAS Molecules: A Major Concern for the Human Health and the Environment. Toxics.

[33] Reardon A.J.F., Hajihosseini M., Dinu I., Field C.J., Kinniburgh D.W., MacDonald A.M., Dewey D., England-Mason G., Martin J.W., APrON Study (2023). Maternal co-exposure to mercury and perfluoroalkyl acid isomers and their associations with child neurodevelopment in a Canadian birth cohort. Environ. Int..

